# Innate-like T Cell Biology in the Tumor Microenvironment Implications for Cancer Immunotherapy

**DOI:** 10.3390/cells15050402

**Published:** 2026-02-26

**Authors:** Maryam Sanjari Pour, Ahmad Nasimian, Julhash U. Kazi

**Affiliations:** 1 Division of Translational Cancer Research, Department of Laboratory Medicine, Lund University, 22363 Lund, Sweden; 2 Lund Stem Cell Center, Department of Laboratory Medicine, Lund University, 22184 Lund, Sweden; 3 Lund University Cancer Centre (LUCC), Lund University, 22184 Lund, Sweden

**Keywords:** innate-like T cells, iNKT, MAIT, γδ T cells, tumor microenvironment (TME), immune surveillance, immune evasion, therapeutic reactivation

## Abstract

**Highlights:**

**What are the main findings?**

- Innate-like T cells, including iNKT, MAIT, and γδ T cells, detect tumor-associated antigenic or stress cues early and support antitumor immunity.
- In established tumors, innate-like T cells are often restrained by reduced CD1d or MR1 antigen presentation, increased inhibitory receptor signaling, suppressive cytokines, and metabolic stress within the tumor microenvironment.

**What are the implications of the main findings?**

- Restoring iNKT, MAIT, and γδ T cell function will likely require matching therapy to the dominant barrier in a given tumor, such as low CD1d or MR1 availability, high checkpoint signaling, suppressive cytokine activity, or metabolic constraint.
- Combination strategies that activate innate-like T cells while reducing local suppression, including agonist-based activation, adoptive or engineered cell platforms, checkpoint blockade combinations, and metabolic interventions, may improve responses in tumors with poor immunotherapy sensitivity.

**Abstract:**

Innate-like T cells (ILTCs) link innate immune responses with adaptive immune functions. This group includes invariant natural killer T (iNKT) cells, mucosa-associated invariant T (MAIT) cells, and γδ T cells. ILTCs detect transformed or stressed cells via non-classical antigen presentation pathways. For example, iNKT cells recognize CD1d-presented glycolipids, MAIT cells respond to MR1-presented metabolites from riboflavin pathways, and γδ T cells sense phosphoantigens through butyrophilin-dependent mechanisms and stress ligands. These features support early tumor control and shape downstream immunity by promoting dendritic cell activation, NK cell function, and priming of tumor-reactive CD8^+^ T cells. In established tumors, ILTC activity is frequently suppressed. Reduced antigen presentation, inhibitory cytokines, hypoxia, and metabolic constraints, including lactate accumulation and kynurenine production, limit effector responses and promote hyporesponsive states. Transcriptional regulators such as TOX, NR4A family members, and BATF are associated with these programs. This review discusses ILTC roles in tumor surveillance, immune escape, and therapeutic strategies to restore their function.

## 1. Introduction

The immune system relies on coordinated cellular and molecular processes to preserve tissue homeostasis and remove infected or transformed cells. Within this framework, T lymphocytes serve as major components of adaptive immunity [1]. Conventional CD4^+^ and CD8^+^ αβ T cells recognize peptide antigens presented by classical major histocompatibility complex (MHC) molecules and generate antigen-specific responses that support immunological memory [2]. Unconventional T cell subsets, often grouped under the term innate-like T cells (ILTCs), have expanded current understanding of immune surveillance [3]. These cells possess rearranged T cell receptors (TCRs), yet they respond rapidly and often without the need for prior antigen exposure, resembling innate immune behavior [4,5].

**Figure 1:**
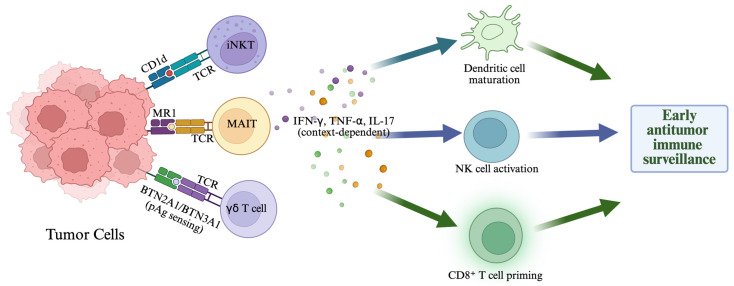
Early tumor sensing by innate-like T cells and coordination of antitumor immunity. Tumor-associated cues engage ITLCs, including iNKT cells, MAIT cells, and γδ T cells. iNKT cells recognize glycolipid antigens presented by CD1d. MAIT cells respond to MR1-presented small-molecule ligands, and human Vγ9Vδ2 T cells can be activated through phosphoantigen-driven signaling mediated by BTN2A1/BTN3A1. Following activation, these cells rapidly release cytokines such as IFN-γ, TNF-α, and IL-17, with the balance of cytokines varying by tissue and tumor context. Through these early effector signals, innate-like T cells promote dendritic cell maturation, support natural killer (NK) cell activation, and facilitate priming of tumor-reactive CD8^+^ T cells, together contributing to early antitumor immune surveillance. Created in BioRender. Uddin K. (2026) https://BioRender.com/7hhqlbs.

ILTCs include invariant natural killer T (iNKT) cells, mucosa-associated invariant T (MAIT) cells, and γδ T cells. These ILTCs are distinctive, as they recognize antigens in ways that do not follow the typical peptide-MHC pathway [6,7,8,9,10]. iNKT cells detect glycolipids presented by CD1d [6,7], MAIT cells respond to riboflavin pathway metabolites presented by MR1 [8,9], and γδ T cells sense phosphoantigens and stress-induced ligands with limited reliance on conventional MHC presentation [10,11]. Such type of recognition biology helps explain their rapid responses. Once activated, they quickly release cytokines such as IFN-γ, TNF-α, and IL-17 [12,13,14]. These early effector functions allow ILTCs to contribute to immune surveillance during infection, cellular stress, and malignant transformation. They also shape broader immune responses by interacting with dendritic cells (DCs), natural killer cells (NKs), and conventional αβ T cells (Figure 1).

### 1.1. ILTCs at the Interface of Innate and Adaptive Immunity

ILTCs sit at the interface between rapid, innate-style defense, and longer-term adaptive immunity. ILTCs shape adaptive immune responses by releasing cytokines and engaging in costimulatory interactions that support antigen presentation and effector T cell activation [15,16]. Through these activities, ILTCs connect early inflammatory signals with the development of sustained adaptive immune responses [15].

Immune surveillance has long centered on cytotoxic CD8^+^ T cells and natural killer cells. ILTCs are also well-involved in this process [17,18]. Their ability to detect stress-related or metabolically altered ligands enables recognition of early tumor-associated changes that standard T cell pathways may miss [18,19,20]. Cytokines produced by ILTCs, including IFN-γ and TNF-α, promote pro-inflammatory macrophage differentiation and improve antigen-presenting activity of dendritic cells, thereby strengthening subsequent adaptive antitumor responses [16,21].

### 1.2. Immune Surveillance and Tumor Control

During early tumor development, ILTCs respond quickly and, in some settings, contribute to tumor control. iNKT cells directly kill CD1d-expressing tumor cells through perforin and granzymes or via Fas–FasL interactions [22,23,24]. They also modulate local immunity through cytokine production. IFN-γ promotes activation of NK cells and macrophages, whereas IL-4 and IL-13 may instead favor type-2 skewed programs that have context-dependent effects within the tumor microenvironment (TME) [25,26,27]. γδ T cells recognize cellular stress or transformation signals, including NKG2D ligands such as MICA/B, and also sense phosphoantigens through butyrophilin family molecules [19,28,29]. MAIT cells, while best known for antimicrobial defense, are present in multiple human tumors. Their association with outcome varies across settings and may depend on local cues that bias their effector profile toward inflammatory or suppressive programs [5,30,31,32].

ILTCs influence tumor surveillance by regulating cellular crosstalk. Their early cytokine release supports myeloid cell recruitment, promotes DC maturation, and improves priming of tumor-reactive CD8^+^ T cells [15,21,33]. Adoptive transfer of MAIT cells restrained tumor growth in a mouse colon cancer model through immune modulation involved eosinophil recruitment and activation, pointing to a MAIT-linked pathway that may contribute to tumor control in selected settings [34]. Taken together, these observations support the view that ILTCs connect early inflammatory sensing with later tumor-directed adaptive responses.

### 1.3. Tumor-Induced Immune Evasion

Although ILTCs utilize durable effector functions, their activity is often constrained as tumors progress. The TME contains multiple suppressive elements, including hypoxia, adenosine, lactic acid, and regulatory cytokines such as IL-10 and TGF-β, which together reduce immune cell responsiveness [21,35,36,37]. Reduced surface expression of CD1d and MR1 on tumor cells may limit their recognition by iNKT and MAIT cells [38,39,40,41]. In human tumor-infiltrating samples from solid tumors, such as hepatocellular carcinoma, colorectal cancer, and non-small cell lung cancer, and in corresponding murine models, chronic stimulation together with suppressive cues has been associated with increased expression of inhibitory receptors and reduced cytokine output in ILTCs [38,40,42,43,44,45].

**Figure 2:**
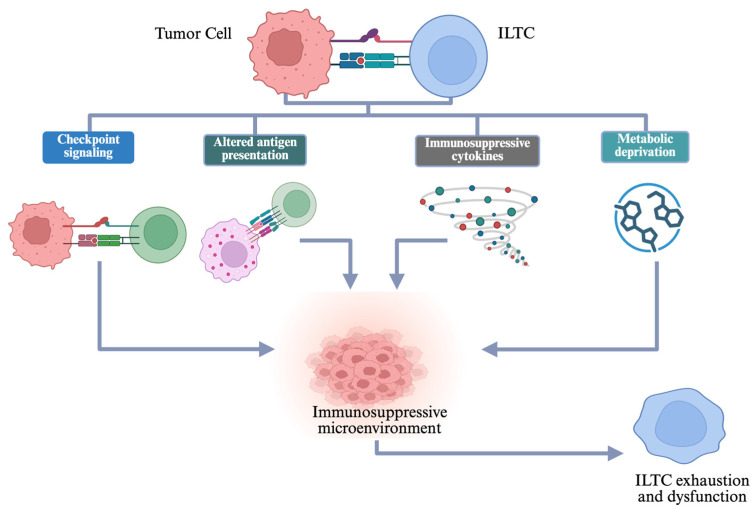
Tumor-mediated evasion of innate-like T cell activity. Tumors blunt ILTC-mediated immunity through inhibitory receptor engagement (PD-1 or PD-L1), reduced non-classical antigen-presentation (CD1d, MR1), secretion of immunosuppressive cytokines (IL-10, TGF-β), and metabolic constraints (hypoxia, lactate accumulation, nutrient depletion). These factors reduce activation, inhibit cytotoxicity, and bias cytokine programs within the TME. Created in BioRender. Uddin K. (2026) https://BioRender.com/7hhqlbs.

Transcription factors are known to drive dysfunctional T cell states in chronic infections and cancer [46,47,48,49,50]. Related transcriptional programs may also operate in innate-like T cell subsets, although the extent and context of their involvement remain incompletely defined. Metabolic constraints further limit ILTC function within tumors. Tumor-derived metabolites and suppressive myeloid cells alter ILTC metabolic pathways, restricting glycolytic flux and mitochondrial activity required for sustained effector responses [51,52]. Clarifying how these immune evasion mechanisms operate will inform strategies aimed at restoring ILTC activity within the TME (Figure 2).

### 1.4. Therapeutic Reactivation and Translational Perspective

ILTCs are being explored as targets for cancer immunotherapy because of their rapid effector capacity and ability to sense metabolic and antigenic changes within tumors. Multiple therapeutic strategies aim to restore or enhance ILTC activity within TME. These include pharmacological stimulation, such as CD1d-binding glycolipid agonists for iNKT cells, MR1-modulating ligands for MAIT cells, and phosphoantigen- or butyrophilin-based activation approaches for γδ T cells [53,54,55,56,57,58,59,60]. In parallel, adoptive transfer approaches, including expansion of endogenous ILTCs and CAR-engineered platforms, are being developed to increase tumor-directed activity and overcome local suppression [55,56,59]. Together, these approaches place ILTCs within a translational framework that links early tumor sensing, mechanisms of dysfunction within the TME, and efforts to restore or redirect antitumor immunity in cancer immunotherapy.

### 1.5. Literature Search Strategy and Selection Criteria

We performed a targeted literature search in PubMed (last searched: November 2025) using combinations of controlled vocabulary and free-text terms related to invariant natural killer T cells (iNKT), mucosal-associated invariant T cells (MAIT), γδ T cells, CD1d, MR1, BTN2A1, BTN3A1, and cancer (e.g., tumor, carcinoma, melanoma, lymphoma, and immunotherapy). Reference lists of relevant reviews and primary studies were screened to capture earlier mechanistic and translational works. No formal meta-analysis was performed; instead, evidence was synthesized in a context-dependent manner that explicitly notes tumor type, compartment, and evidence source when interpreting functional states. Later, some recent references have been added during the revision.

## 2. Subsets of ILTCs and Their Semi-Invariant TCRs

TCR repertoires of ILTCs are often constrained relative to conventional αβ T cells, with semi-invariant or biased usage that supports rapid responses to conserved microbial products or stress-associated cues. This section summarizes key ILTC subsets and their characteristic TCR features and briefly notes additional innate-leaning αβ T cell populations, including CD8αα intraepithelial lymphocytes and cytokine-preactivated effector-memory T cells (Table 1). Across studies, ILTC frequency and phenotype differ between peripheral blood, tumor tissue, draining lymph nodes, and adjacent non-malignant tissues, and they vary with tumor histology, anatomical site, and prior therapy.

### 2.1. iNKT Cells

iNKT cells are characterized by expression of a semi-invariant TCR composed of an invariant α-chain (Vα14–Jα18 in mice and Vα24–Jα18 in humans) paired with a restricted set of β-chains (Vβ8.2, Vβ7, or Vβ2 in mice and Vβ11 in humans) [61,62]. This TCR recognizes glycolipid antigens presented by the monomorphic CD1d molecule [63,64,65]. The prototypical synthetic ligand, α-galactosylceramide, activates iNKT cells and induces rapid secretion of cytokines, including IFN-γ, IL-4, IL-13, and GM-CSF [66,67,68].

Following activation, iNKT cells influence tumor immunity through both direct and indirect mechanisms [69]. They are capable of lysing CD1d-expressing tumor cells through perforin- and granzyme-dependent pathways. In parallel, iNKT-derived IFN-γ supports the activation of natural killer cells, macrophages, and CD8^+^ CTLs, while interactions with dendritic cells promote IL-12 production and maturation of antigen-presenting cells [70,98]. Mature dendritic cells upregulate costimulatory molecules such as CD80 and CD86, further enhancing Th1-biased immunity [99,100].

Functionally, iNKT cells are subdivided into populations analogous to helper T cell lineages, including NKT1, NKT2, and NKT17 cells. NKT1 cells predominantly produce IFN-γ, NKT2 cells secrete IL-4 and IL-13, and NKT17 cells generate IL-17A and IL-22 [67,101,102,103]. The distribution of these subsets is regulated by local cytokines, tissue environment, and developmental cues. In tumor settings, enrichment of IFN-γ-producing NKT1 cells has been associated with improved clinical outcomes, whereas polarization toward NKT2 or NKT17 programs has been linked to immune suppression in certain contexts [104,105]. Single-cell transcriptomic and chromatin accessibility studies have further revealed functional diversity within the iNKT compartment, including IL-10-producing NKT10 cells and tissue-adapted populations in organs such as adipose tissue and liver, which regulate local inflammatory tone [106,107,108,109,110].

### 2.2. MAIT Cells

MAIT cells form another evolutionarily conserved T cell population with innate-like properties. Their semi-invariant TCR (Vα7.2–Jα33 in humans and Vα19–Jα33 in mice) pairs predominantly with a set of TCR β chains (Vβ2 and Vβ13) and recognizes vitamin B_2_ (riboflavin)-derived metabolites presented by the MHC-related molecule MR1 [8,72,73,111,112,113]. This antigen presentation pathway enables MAIT cells to detect a broad range of microbial and metabolic ligands independent of peptide–MHC recognition [114].

Upon activation, MAIT cells secrete cytokines, including IFN-γ, TNF-α, and IL-17, and exert cytotoxicity via perforin and granzyme B [74,75]. They are enriched at mucosal barriers such as the gut, lungs, and liver, where they contribute to immune homeostasis and tumor surveillance [76,77,78,115]. In cancer, MAIT cells exhibit context-dependent duality. When polarized toward a Th1-like profile, MAIT-derived IFN-γ and TNF-α support antitumor activity [116,117]. However, chronic exposure to tumor-derived metabolites and inhibitory cytokines (IL-10, TGF-β) drive them toward IL-17-dominant, pro-tumoral phenotypes. High tumor infiltration by IL-17-producing MAIT cells correlates with poor prognosis in human hepatocellular carcinoma and colorectal cancer cohorts [44,75,118,119].

### 2.3. γδ T Cells

γδ T cells represent the most evolutionarily conserved and functionally diverse ILTC subset. Unlike αβ T cells, they recognize phosphoantigens and stress-induced self-ligands independently of classical MHC presentation [39,120,121]. Human γδ T cells are classified mainly into Vδ1, Vδ2, and Vδ3 subsets based on TCR δ-chain usage [81,82].

The Vγ9Vδ2 population, predominant in peripheral blood, senses metabolic dysregulation through phosphoantigens derived from the mevalonate and non-mevalonate pathways such as isopentenyl pyrophosphate (IPP) and (E)-4-hydroxy-3-methyl-but-2-enyl pyrophosphate (HMBPP) in a butyrophilin (BTN3A1/BTN2A1)-dependent manner [82,83,84,85]. In contrast, epithelial-resident Vδ1 cells respond to stress-induced molecules such as MICA/B and UL16-binding proteins (ULBPs), contributing to tissue repair and tumor surveillance [120,121,122,123].

Upon activation, γδ T cells rapidly release cytotoxic mediators (perforin, granzyme B, and TNF-α) and proinflammatory cytokines such as IFN-γ and IL-17 [86]. Notably, IFN-γ-dominant Vγ9Vδ2 cells promote antitumor immunity, whereas IL-17-producing Vδ1 cells may support angiogenesis and tumor progression in certain contexts [124,125].

Collectively, iNKT, MAIT, and γδ T cells integrate innate pattern recognition with adaptive receptor specificity, forming a rapid-response network essential for early tumor immunosurveillance and immune regulation [126,127]. Single-cell RNA-seq and TCR-seq have revealed remarkable diversity in γδ T cell repertoires, uncovering effector subsets with stem-like and exhausted phenotypes depending on tissue context [80,86,87,128]. These findings highlight their plasticity and potential for therapeutic exploitation.

### 2.4. Innate-like αβ T Cells and Other Subsets

Several αβ T cell populations also display innate-like behavior [88]. Examples include CD8αα intraepithelial lymphocytes (IELs), H2–M3-restricted T cells, and semi-invariant tissue-resident memory T cells (Trm) that respond to cytokines (IL-12, IL-18) independent of TCR ligation [3,89,90,91,92,93,94]. These subsets display transcriptional profiles enriched for PLZF, Hobit, and Blimp-1, endowing them with pre-armed effector potential and the ability to rapidly release cytokines upon activation [95,96,97].

## 3. Tumor Surveillance and Antitumor Functions

ILTCs serve as mediators of immune surveillance, linking early innate detection of malignant transformation with adaptive immune responses [129]. Unlike conventional αβ T cells, which rely on peptide–MHC recognition, as discussed above, ILTCs sense tumor-associated changes through non-classical antigen presentation systems. These recognition mechanisms allow them to identify lipid antigens, microbial metabolites, or stress-induced self-ligands that accumulate during oncogenic transformation and position them as early sentinels of tumorigenesis [15,129].

### 3.1. iNKT Cells in Tumor Surveillance

NKT cells are one of the best-studied innate-like T cell subsets in cancer immunity, and they influence tumor control through direct killing and through regulation of other immune cells. In settings where tumor cells express CD1d, iNKT cells recognize these targets and induce cell death through perforin and granzymes or via Fas–FasL pathways [71,130,131]. Activated iNKT cells also produce IFN-γ, which supports cytotoxic activity of NK cells and CD8^+^ cytotoxic T lymphocytes (CTLs). IFN-γ, together with contact-dependent interactions, promote DC maturation and improve antigen presentation, supporting downstream adaptive responses [130,132,133].

Beyond cytokine-mediated support, iNKT cells further amplify antitumor immunity through IL-12-producing DCs and costimulatory interactions involving NKG2D ligands and CD70 expressed on DCs. All these interactions promote CTL priming and memory formation, crucial for maintaining antitumor immunity and preventing relapse [71,130,131].

Experimental models demonstrate accelerated tumor growth and metastasis in iNKT-deficient mice, underscoring their essential role in immune surveillance [15]. Clinically, higher iNKT infiltration correlates with improved outcomes in melanoma, head and neck cancer, and hepatocellular carcinoma [134,135,136].

### 3.2. MAIT Cells and Tumor Recognition

MAIT cells recognize tumor-associated metabolic alterations via MR1-mediated presentation of riboflavin-derived intermediates [8,58,137]. Upon activation, they rapidly produce IFN-γ, TNF-α, and granzyme B, supporting antitumor immunity [79,138,139]. However, their functional outcome within the tumor TME appears highly context-dependent. Microbiome-responsive MAIT signatures add another layer of context dependence [140,141]. During early tumorigenesis, MAIT cells display Th1-like, cytotoxic phenotypes that support tumor control. Whereas chronic antigenic stimulation and exposure to IL-10, TGF-β, or hypoxic conditions polarize them toward IL-17-producing, pro-tumoral phenotypes [4,117].

Clinical observations reveal that the frequency and cytokine profile of MAIT cells differ between peripheral blood and tumor sites. For instance, reduced circulating MAIT cells but increased tumor infiltration in colorectal and hepatocellular carcinoma are patterns frequently associated with disease progression [45,119]. Elucidating the determinants of this functional duality remains a major research priority.

### 3.3. γδ T Cells as Rapid Cytotoxic Responders

γδ T cells constitute a lymphocyte compartment capable of rapid recognition of transformed or stressed cells without reliance on classical MHC restriction. Their activation is triggered by phosphoantigens and stress-associated ligands sensed through butyrophilin (BTN and BTNL)-regulated pathways, enabling prompt responses during tumor development [82,120,121,122,123]. Within human γδ T cells, the Vγ9Vδ2 subset has been studied extensively for its ability to detect metabolic alterations in tumor cells. Dysregulation of the mevalonate pathway leads to accumulation of phosphoantigens that activate Vγ9Vδ2 T cells in a BTN-dependent manner [82,142]. Upon activation, these cells secrete IFN-γ and TNF-α and release cytotoxic granules containing perforin and granzymes [143]. This functional plasticity highlights the importance of tissue and environmental context in shaping γδ T cell responses.

In breast, lung, and colorectal cancer cohorts, γδ T cell infiltration has been linked to either favorable or unfavorable outcomes depending on whether cytotoxic IFN-γ-skewed programs (often Vγ9Vδ2-like) or IL-17-skewed programs predominate in tumor tissue [142,143,144]. However, the functions of γδ T cells are not uniformly protective; certain γδ subsets, such as IL-17-producing Vδ1 cells, promote angiogenesis and inhibit DC maturation, facilitating immune escape and tumor progression [82]. This dual nature underscores the functional plasticity of γδ T cells and highlights the importance of understanding their context-dependent roles in the tumor microenvironment.

### 3.4. Cooperative Networks in Immune Surveillance

ILTCs operate within interconnected immune circuits rather than as isolated effector populations. Through cytokine secretion and receptor–ligand interactions, ILTC subsets coordinate multiple arms of the immune response to optimize tumor surveillance. For instance, iNKT cell-derived IFN-γ enhances NK cell cytotoxicity and promotes the activation of γδ T cells, reinforcing early antitumor responses [82,144]. MAIT and γδ T cells, in turn, modulate macrophage polarization toward M1-like, proinflammatory phenotypes and promote DC maturation, thereby strengthening antigen presentation and CD8^+^ T cell priming [122,123,145].

These cooperative networks enable ILTCs to function as immune amplifiers, linking stress detection and metabolic sensing to adaptive effector activation [24,82,116]. However, under conditions of chronic antigen exposure, metabolic restriction, or hypoxia within the TME, these communication circuits can be disrupted and result in dysfunctional cytokine signaling and impaired surveillance capacity [21,35,36,37,38,39,40,41,42,43,46,47,48,49,50,51,52]. Restoring these intercellular circuits may therefore be critical for reestablishing effective antitumor immunity, as discussed in the following section.

## 4. Immune Evasion and Functional Dysregulation

Although ILTCs possess substantial antitumor potential, tumors frequently evolve multifaceted strategies that impair their function and escape immunological control. Persistent antigenic stimulation, metabolic deprivation, hypoxia, and suppressive cytokine signaling within the TME drive these cells toward exhaustion and functional paralysis [46,47,48,49,50,51,52]. Within the TME, these mechanisms act in concert, dampening activation signals, altering metabolism, and reprogramming gene expression, to drive ILTCs into states of exhaustion and dysfunction. Understanding how these layers of suppression converge is essential to deciphering the balance between tumor control and immune escape.

### 4.1. Loss of Antigenic Visibility: Downregulation of CD1d and MR1

One of the most direct and early routes by which tumors avoid ILTC-mediated recognition is through reducing the molecular “visibility” of non-classical antigen presentation. iNKT and MAIT cells rely on the presentation of lipid or metabolite antigens via CD1d and MR1, respectively. However, multiple solid malignancies, including hepatocellular carcinoma, melanoma, and certain breast cancers, demonstrate marked downregulation of CD1d, thereby preventing effective iNKT engagement and killing [44,55,146,147].

Similarly, MR1 expressions can be suppressed by metabolic and hypoxic cues within the TME. This interferes with the loading of riboflavin-derived intermediates critical for MR1 surface presentation, which effectively dampen MAIT-cell activation [148,149]. Reduced non-classical antigen presentation impairs the ability of ILTCs to recognize transformed cells, thereby enabling tumor immune evasion and sustained growth.

### 4.2. Cytokine-Mediated Suppression and the Metabolic Trap

Within tumors, iNKT cells often have profound reductions in cytotoxicity and cytokine secretion as a result of chronic antigen exposure and the sustained engagement of inhibitory receptors such as PD-1, TIM-3, and LAG-3 [150,151,152]. These signals collectively induce a state of functional exhaustion characterized by impaired IFN-γ secretion, reduced proliferative capacity, and increased apoptotic susceptibility. Beyond inhibitory receptors, tumor-derived lipids interfere with CD1d-mediated antigen presentation, while regulatory dendritic cells secrete IL-10 and TGF-β, further dampening iNKT activation and skewing their function toward tolerogenic phenotypes [153,154,155].

Persistent exposure to immunosuppressive cytokines such as IL-10 and TGF-β exacerbates this effect by driving functional reprogramming of ILTCs, transforming formerly IFN-γ-producing cytotoxic subsets into IL-17- or IL-10-secreting populations that favor immunosuppression rather than tumor clearance [117]. This cytokine-driven polarization is tightly coupled to metabolic stress: elevated tumor glycolysis leads to lactic acid accumulation, while IDO-mediated tryptophan catabolism generates immunosuppressive kynurenine metabolites that inhibit mTOR signaling and suppress effector granule release [15,156,157].

Through these parallel suppressive pathways, cytokine-mediated signaling, inhibitory receptor engagement, and metabolic deprivation, the TME creates a coordinated metabolic trap that renders ILTCs progressively less capable of sustaining antitumor activity.

### 4.3. Functional Exhaustion and Transcriptional Rewiring

Unconventional T cell subsets, including iNKT, MAIT, and γδ T cells, are increasingly recognized to adopt dysfunctional or exhausted phenotypes in the TME [40]. These cell types, normally positioned at the intersection of innate and adaptive immunity, exhibit a characteristic exhaustion profile marked by high expression of inhibitory receptors (PD-1, TIM-3, LAG-3, and CD39), alongside diminished secretion of effector cytokines such as IFN-γ and TNF-α and impaired cytotoxic activity.

At the transcriptional level, chronic stimulation within tumors or inflammatory niches drives NFAT-dependent induction of transcriptional repressors, including TOX and the NR4A family. These factors orchestrate broad chromatin remodeling events that restrict access to AP-1-dependent effector loci while stabilizing inhibitory gene networks. Although the core exhaustion circuitry shares similarities with that of conventional CD8^+^ T cells, iNKT, MAIT, and γδ T cells, it exhibits lineage-specific rewiring shaped by its innate-like transcriptional and metabolic programs. This rewiring not only alters effector and memory-like differentiation but also constrains their responsiveness to immunotherapeutic interventions [40]. As a result, ILTC exhaustion represents a major barrier to durable antitumor immunity. Clarifying the molecular and metabolic mechanisms underlying this dysfunctional state is essential for designing strategies to reinvigorate these unconventional T cell populations within the TME.

## 5. Therapeutic Reactivation and Translational Potential

**Figure 3:**
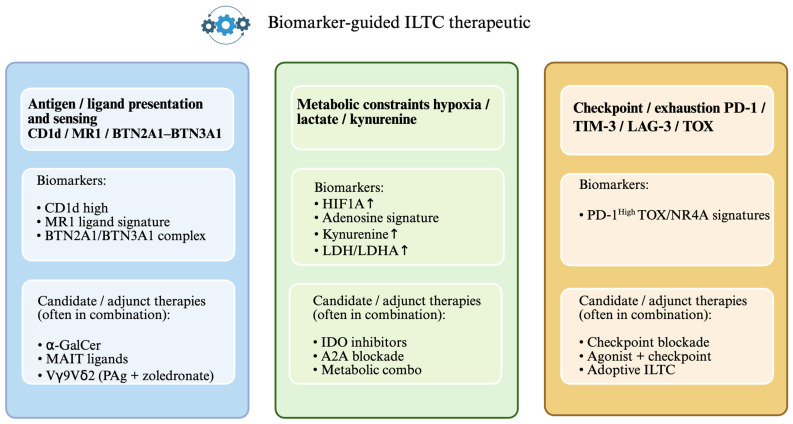
Biomarker-guided ILTC therapeutic strategies. Presence of CD1d, MR1 ligand signature, or the BTN2A1/BTN3A1 complex suggests suitability for engaging iNKT, MAIT, or Vγ9Vδ2 γδ T cell pathways, respectively, motivating use of agonists such as α-GalCer (iNKT), MAIT ligands, or phosphoantigen/aminobisphosphonate-based activation (e.g., PAg + zoledronate) for Vγ9Vδ2 cells. Biomarkers of hypoxia (e.g., HIF1A), adenosine signaling, kynurenine, and lactate-associated glycolysis (e.g., LDH/LDHA) indicate a suppressive metabolic tumor microenvironment and support adding metabolic interventions such as IDO pathway inhibition, A2A receptor blockade, or broader metabolic combination strategies. A checkpoint/exhaustion-associated program (e.g., PD-1^high^ with TOX/NR4A signatures) supports the use of checkpoint blockade, agonist + checkpoint combinations, and/or adoptive ILTC approaches. Up arrow indicates increased expression or activity. Created in BioRender. Uddin K. (2026) https://BioRender.com/7hhqlbs.

Given their strategic position at the interface of innate and adaptive immunity, ILTCs have emerged as compelling targets for therapeutic reactivation in cancer (Figure 3). Their ability to respond rapidly, sense metabolic stress, and orchestrate immune crosstalk places them in a unique position to complement or enhance current immunotherapeutic modalities. A summary of major therapeutic strategies targeting ILTCs is provided in Table 2. Reactivating these cells requires strategies that address both their functional potential and the suppressive constraints imposed by the TME. Multiple therapeutic avenues have been explored, ranging from pharmacologic stimulation and engineered cell therapies to checkpoint inhibition and metabolic or epigenetic modulation.

### 5.1. From Mechanisms to Treatment Selection

A practical way to translate ILTC biology into trial design is to match the intervention to the dominant barrier present in an individual tumor. Low CD1D or MR1 expression, loss of antigen-presenting cells, or disrupted BTN2A1 or BTN3A1 signaling suggests that agonists may require delivery via professional antigen-presenting cells, that therapies may need to increase antigen visibility, or that Vγ9Vδ2-directed approaches may need pharmacologic phosphoantigen amplification [58,84,167,168]. High IL-10 or TGF-β activity, strong adenosine or lactate signatures, or enriched IDO1 or ARG1 programs suggest pairing ILTC activation with approaches that attenuate these suppressive pathways [169,170,171,172]. When tumor-infiltrating ILTCs exhibit high checkpoint expression (PD-1, TIM-3, and LAG-3) and reduced cytotoxic programs, a rational strategy is to combine ILTC activation or adoptive transfer with checkpoint blockade while monitoring cytokine polarization so that IL-17-skewed programs are detected early [54,104,157]. This barrier-based framework also supports clearer endpoint selection, including pre-defined immune monitoring for expansion, persistence, trafficking, and cytokine bias in blood and tumor tissue.

### 5.2. Pharmacologic Activation

One of the earliest approaches involves direct stimulation of ILTCs through synthetic ligands. α-galactosylceramide, an activator of iNKT cells, induces robust IFN-γ-driven immune cascades capable of amplifying downstream NK and CD8^+^ T cell responses [15,23,173]. The iNKT agonist α-GalCer, described by Kawano et al. [174], remains the prototype for pharmacologic activation. Newer formulations, including α-GalCer analogs with improved pharmacokinetics and DC-targeted delivery systems, have been developed to enhance iNKT activation while minimizing anergy [175,176,177,178,179,180]. Parallel efforts aim to pharmacologically activate MAIT and γδ T cells. MR1-binding small molecules capable of modulating microbial metabolite presentation have opened avenues for targeted MAIT-cell stimulation, while phosphoantigen analogues and butyrophilin agonists selectively activate γδ T cells, particularly the Vγ9Vδ2 subset [41,58,181,182]. These agents can be administered alone or combined with vaccines, or checkpoint inhibitors, generating broader and more sustained antitumor responses [23,183,184,185].

### 5.3. Adoptive Cell Therapy

Adoptive transfer approaches have brought ILTCs into active translational development. iNKT cells, MAIT cells, and γδ T cells can be expanded ex vivo and infused, and early-phase studies and preclinical models suggest that such products mediate cytotoxic activity with an acceptable safety profile in selected settings [41,82,143,186,187,188,189]. Reported clinical activity varies by platform, disease type, and manufacturing strategy and remains under evaluation in larger studies.

Engineered ILTC products are also being developed. CAR-modified iNKT and γδ T cells, as well as related innate-like platforms, are of interest because they combine engineered target recognition with reduced reliance on classical MHC restriction. Compared with conventional αβ CAR-T cells, several ILTC-based CAR approaches are expected to carry a lower risk of graft-versus-host disease, particularly in allogeneic settings, and may retain complementary modes of tumor recognition through their endogenous receptors [33,36,143,145,190,191,192].

### 5.4. Checkpoint Blockade and Combinatorial Strategies

Immune checkpoint inhibition represents another approach to recover ILTC activity in tumors. Blockade of inhibitory receptors such as PD-1, TIM-3, or LAG-3 has been shown in experimental systems to partially reverse dysfunctional states in iNKT, MAIT, and γδ T cells, leading to improved cytokine production, proliferative capacity, and cytotoxic function [193,194,195,196,197]. These effects are often incomplete when checkpoint blockade is used alone, reflecting the layered suppression present within the TME.

Combining checkpoint inhibitors with ILTC-directed activation strategies has therefore gained interest. In preclinical models, PD-1 blockade combined with α-GalCer-loaded DC vaccination results in stronger and more sustained antitumor responses than either approach alone [23,36,198]. Similar combination concepts are being explored with γδ T cell agonists and MAIT-modulating agents. Such strategies parallel combination approaches developed for conventional αβ T cells while taking advantage of the rapid response kinetics and stress-sensing properties of ILTCs.

### 5.5. Metabolic and Epigenetic Modulation

Given the profound metabolic restrictions imposed by the TME, targeting ILTC metabolism has become a promising therapeutic direction. Strategies aimed at restoring mitochondrial fitness, enhancing glycolytic flexibility, or relieving lactic acid-induced inhibition can reinvigorate ILTC effector functions [52,199,200]. These strategies collectively represent the reactivation arm of the ILTC–tumor axis aimed at converting suppressed or exhausted cells into active antitumor effectors [201,202,203].

Although most mechanistic insights into exhaustion-associated transcriptional regulators such as TOX, NR4A, and BATF come from studies in chronically stimulated CD8^+^ T cells, the emerging [48,204,205] evidence indicates that similar NFAT-driven epigenetic programs may operate in iNKT, MAIT, and γδ T cells as they acquire dysfunctional phenotypes within tumors. These shared transcriptional circuits suggest that targeting exhaustion-associated regulators, originally defined in CD8^+^ T cell biology, may also offer opportunities to reset exhaustion pathways and dysfunctional phenotypes in ILTCs.

### 5.6. Risks, Context-Specific Pro-Tumor Programs, and Safety Considerations

ILTC-based interventions yield opposing outcomes depending on tumor context and the dominant effector program. IL-17-biased MAIT and γδ T cells have been associated with neutrophil recruitment, angiogenesis, and suppressive myeloid programs in some tumors, so non-selective activation may be counterproductive in settings where these programs dominate. Repeated stimulation of iNKT cells with α-GalCer induce a hyporesponsive state and shift cytokine balance; dosing schedule and agonist format (for example, delivery via professional APCs or use of analogs) should therefore be considered during trial design. Tumors evade antigen-dependent activation through reduced CD1d or MR1 expression on malignant cells and/or local antigen-presenting cells, limiting the activity of free agonists in the absence of effective presentation. For adoptive cell products, heterogeneity in starting subsets (for example, Vγ9Vδ2 versus Vδ1 or MAIT subsets) alter expansion, trafficking, and effector output, so product specifications should include phenotypic and functional release criteria. Engineered ILTC therapies also share safety considerations with other cellular immunotherapies, including cytokine release and off-tissue recognition, particularly for receptors that bind stress ligands that can be induced on inflamed non-malignant tissues.

## 6. Conclusions and Future Perspectives

ILTCs occupy a unique niche at the interface of innate and adaptive immunity, yet many aspects of their biology remain incompletely defined. The developmental origins of human ILTC subsets, their tissue-specific adaptations, and the mechanisms governing their crosstalk with the TME continue to be active areas of investigation [5,15,203]. Despite their well-documented antitumor potential, ILTCs exhibit remarkable functional plasticity capable of exerting either protective or tolerogenic effects depending on context underscoring the need for precise therapeutic modulation.

Recent advances in single-cell multi-omics, including scRNA-seq, scATAC-seq, TCR-seq, and spatial transcriptomics, have transformed our understanding of ILTC diversity. These technologies reveal that ILTCs are not static lineages but dynamic, plastic populations that adapt to tissue and metabolic cues [206].

Multi-omic mapping of human liver and lungs has uncovered distinct spatial clusters of MAIT and iNKT cells with IFN-γ-dominant, IL-17-dominant, and regulatory modules [128,207,208]. Similarly, scATAC-seq analyses of murine iNKT cells identified tissue-specific enhancer landscapes correlating with functional fate decisions [109,209,210].

From a translational perspective, harnessing this plasticity represents both a challenge and an opportunity. Integrating ILTC biology into clinical immunotherapy—through strategies such as combining CAR-engineered or pharmacologically-activated ILTCs with checkpoint blockade or metabolic modulators—may enable reactivation of antitumor responses even in immunologically “cold” tumors that resist conventional therapies [162,202,211,212,213,214]. Future research aimed at deciphering the molecular circuits of ILTC activation, exhaustion, and reprogramming will be pivotal to transforming these insights into effective interventions. Together, these findings emphasize the plasticity and adaptability of ILTCs’ properties that make them both challenging and promising targets for next-generation immunotherapies.

Across available datasets, the direction of ILTC function is context-dependent: cytotoxic, IFN-γ-skewed programs dominate in some tumors and accompany immune activation, whereas IL-17-skewed or regulatory programs dominate in others and accompany disease progression. Clinical development of ILTC-targeting approaches should therefore couple intervention choice to measurable tumor features (for example, CD1D or MR1 expression, BTN2A1 or BTN3A1 pathway integrity, inhibitory receptor burden, and cytokine or metabolic signatures) and should predefine immune endpoints such as expansion and persistence of the relevant subset, tumor trafficking, shifts in cytokine bias (IFN-γ versus IL-17), and changes in suppressive myeloid activity.

In conclusion, ILTCs represent a dynamic and versatile component of the immune landscape. Their capacity to sense transformed cells rapidly, orchestrate cross-talk among immune compartments, and be therapeutically re-engaged places them at the center of the continuum linking immune surveillance, immune evasion, and therapeutic reactivation [215,216]. Elucidating the mechanisms that regulate their activation, exhaustion, and reprogramming will open new horizons for next-generation cancer immunotherapy [202,203].

## Figures and Tables

**Table 1:** Classification and key features of ILTCs.

ILTC Subset	Canonical Receptor or TCR Usage (Human)	Restriction and Primary Cue	Cytokines Often Reported (Context-Dependent)	Cytotoxic Mediators	Note
iNKT cells	Semi-invariant TCR (Vα24-Jα18; Vβ11)	CD1d-presented glycolipids (α-GalCer and endogenous glycolipids)	IFN-γ, TNF, IL-4; IL-17 in NKT17 programs	Perforin, granzymes, FasL, TRAIL	Frequency and function differ between blood and tumor [61,62,63,64,65,66,67,68,69,70,71].
MAIT cells	Semi-invariant TCR (Vα7.2-Jα33/12/20 with limited Vβ usage)	MR1-presented small-molecule ligands (riboflavin pathway metabolites; MR1-binding synthetic molecules)	IFN-γ, TNF; IL-17 in some tumor settings	Perforin, granzymes	Often enriched in mucosal tissues and liver [8,58,72,73,74,75,76,77,78,79].
γδ T cells	Vγ9Vδ2 common in blood; Vδ1 or Vδ3 enriched in tissues	Vγ9Vδ2 activation via phosphoantigen-driven BTN2A1 and BTN3A1 signaling; Vδ1 responds to stress ligands and lipid antigens (often with NK receptors such as NKG2D)	IFN-γ, TNF; IL-17 in subsets and tumor contexts	Perforin, granzymes, granulysin	Polarization differs by subset, tissue site, and tumor type [80,81,82,83,84,85,86,87].
Innate-like αβ T cells or IEL-like programs	Tissue-resident αβ T cells with rapid effector capacity (e.g., CD8αα IEL-like programs)	Non-classical MHC I in humans (e.g., HLA-E) plus stress signals; NK receptor engagement	IFN-γ, TNF; tissue-repair cytokines in some contexts	Perforin, granzymes	Mainly tissue-resident; cancer evidence is tissue-specific [3,88,89,90,91,92,93,94,95,96,97].

**Table 2:** Selected ILTC-focused therapeutic approaches (clinical studies and preclinical concepts).

Evidence Level	Strategy or Platform	Target ILTC Subset	Example Indication or Setting	Registry Identifier	Phase or Design	Notes
Clinical	Systemic α-GalCer (KRN7000) administration	iNKT	Refractory solid tumors	NCT00003985	Phase I; withdrawn	Withdrawn prior to enrolling any participants.
Clinical	α-GalCer-pulsed dendritic cells (Chiba-NKT)	iNKT	Advanced or recurrent NSCLC	UMIN000007321	Phase II; single arm; target n = 35	As a second-line therapy, prolonged overall survival [53].
Clinical	α-GalCer-pulsed DCs plus activated NKT infusion	iNKT	Head and neck squamous cell carcinoma	UMIN000000722	Phase I/II; single arm; target n = 14	Eight patients were enrolled and showed significant antitumor immunity [158].
Clinical	α-GalCer-pulsed APC adjuvant after radiotherapy	iNKT	Head and neck mucosal melanoma	UMIN000009430	Phase II; randomized, double-blind; target n = 50	Unpublished results.
Clinical	Zoledronic acid plus low-dose IL-2 to expand Vγ9Vδ2 cells	γδ T (Vγ9Vδ2)	Post-haplo-identical allogeneic SCT	NCT03862833	Phase I; completed; n = 26	A clinical example of in vivo expansion [159].
Clinical	Allogeneic NKG2DL-targeting CAR γδ T cells (CTM-N2D)	γδ T	Advanced cancers	NCT05302037	Phase I; dose escalation	Ongoing early-phase evaluation [160].
Clinical	Allogeneic CAR γδ T cells CAR001 (HLA-G-CAR.BiTE γδ)	γδ T	Relapsed or refractory solid tumors (NSCLC, TNBC, CRC, GBM)	NCT06150885	Phase I/IIa; target n = 60	Early phase.
Clinical	GD2-CAR plus IL-15-armored iNKT cells (GINAKIT2)	iNKT (engineered)	Relapsed or refractory neuroblastoma	NCT03294954	Phase I; dose escalation: target n = 70	Expansion and safety of CAR-NKT [161,162,163,164].
Preclinical	MR1 ligand delivery (e.g., 5-OP-RU analogs) to activate MAIT cells	MAIT	Solid tumor models		Experimental	A preclinical study [165].
Preclinical	Adoptive MAIT transfer or engineered MAIT products	MAIT	Mouse tumor models		Experimental	Subset selection [166].

## Data Availability

No new data were created or analyzed in this study.
